# Ciguatera Fish Poisoning, Canary Islands

**DOI:** 10.3201/eid1112.050393

**Published:** 2005-12

**Authors:** Jose-Luis Pérez-Arellano, Octavio P. Luzardo, Ana Pérez Brito, Michele Hernández Cabrera, Manuel Zumbado, Cristina Carranza, Alfonso Angel-Moreno, Robert W. Dickey, Luis D. Boada

**Affiliations:** * University of Las Palmas de Gran Canaria, Las Palmas de Gran Canaria, Spain; † Hospital Universitario Insular de Gran Canaria (Canary Health Service), Las Palmas de Gran Canaria, Spain; ‡ Hospital de Fuerteventura, Puerto del Rosario, Spain; § Gulf Coast Seafood Laboratory (Food and Drug Administration), Dauphin Island, Alabama, USA

**Keywords:** ciguatera fish poisoning, Canary Islands, West African cOSAT

**To the Editor:** Ciguatera outbreaks usually occur in the area between 35° north and 35° south latitude, mainly in the Caribbean, Indo-Pacific islands, and the Indian Ocean (*1**–**5*) (Figure). Occasionally, ciguatera poisoning has been reported outside disease endemic areas, such as the Bahamas, Canada, or Chile, but no case had been described in the West African region until now. European and Spanish cases have been rarely described and are mainly associated with seafood imported from disease-endemic regions (*6*).

**Figure:**
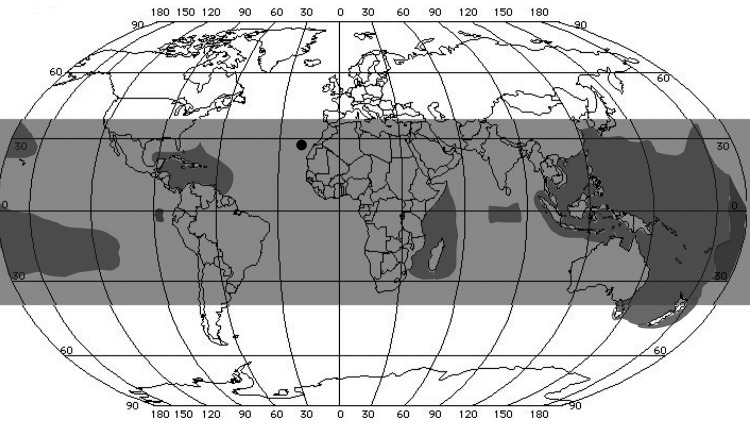
Worldwide distribution of ciguatera. Gray indicates coral reef regions located between 35° north and 35° south latitudes; brown indicates disease-endemic areas of ciguatera; red circle indicates Canary Islands (latitude 28°06´ north, longitude 15°24´ west. Source: refs 4. and 5.

Ciguatera fish poisoning is a clinical syndrome caused by eating contaminated fish (*1*). The causative toxins of its clinical manifestations are ciguatoxins (*7*). These toxins are transmitted by dinoflagellates of the species *Gambierdiscus toxicus*, which lives adhered to damaged coral reefs in tropical seas (*2*). Herbivorous fish species accumulate toxins in their musculature, liver, and viscera after ingesting dinoflagellates. Larger marine carnivores eat contaminated fish and concentrate ciguatoxins (*1**,**2*).

More than 425 species of fish are associated with ciguatera poisoning in humans. The most commonly implicated fish are barracuda, red snapper, grouper, amberjack, sea bass, surgeonfish, and moray (eel) (*2**,**3*). In January 2004, 2 fishermen captured a 26-kg amberjack (local name: Medregal Negro; scientific name: *Seriola Rivoliana*) while scuba diving along the coast of the Canary Islands, Spain. The fishermen filleted the fish and stored fillets in a household freezer. Within a few days, one of the fishermen and 4 family members consumed some fish, and neurologic and gastrointestinal symptoms developed within 30 minutes to 28 hours. The 5 family members sought treatment at the emergency room of Hospital de Fuerteventura and the Outpatient Clinic of Infectious Diseases and Tropical Medicine Service of Hospital Insular de Las Palmas.

The 5 family members exhibited a combination of gastrointestinal (diarrhea [4 persons], nausea/vomiting [3 persons], metallic taste [1 person]), cardiologic (heart rhythm disturbances [2 persons]), systemic (fatigue [5 persons], itching [3 persons], dizziness (1 person]), and neurologic manifestations (myalgia [3 persons], peripheral paresthesia [3 persons], perioral numbness [2 persons], and reversal of hot and cold sensations [3 persons], which is pathognomonic of ciguatera poisoning). These clinical observations and laboratory data were collected from a prospective questionnaire filled in by physicians at the patients' first visits. No hematologic or biochemical abnormalities were detected in any patient. Based upon the symptomatic profiles, relationships of the patients, and their common dietary histories, ciguatera intoxication was diagnosed in all. None of the patients required hospitalization. The neurologic and gastrointestinal symptoms resolved over several weeks, but intermittent recurrence of some symptoms, at lower intensities, was noted for several months.

A portion of the implicated fish was recovered from freezer storage at the fisherman's home. A solid-phase membrane immunobead assay with a monoclonal antibody directed against Pacific ciguatoxins and related polyether toxins was used to detect ciguatoxins or other antigenically related substances in fish tissues. Results were positive.

A 150-g sample of the fish was delivered to the US Food and Drug Organization's Gulf Coast Seafood Laboratory, Dauphin Island, Alabama, USA, for sodium channel–specific in vitro assay (*8*) and liquid chromatography–mass spectrometry (LC/MS/MS) analysis. Assay results were positive and the ciguatoxin content of the fish sample was estimated to be 1.0 ppb (ng/g). Caribbean ciguatoxin (CCTX-1: MH+ m/z 1141.6) was confirmed by LC/MS/MS by using multiple reaction monitoring (*9*). The amount of ciguatoxin in the fish tissue estimated by in vitro assay was low, and close to the limit the LC/MS/MS method can detect. At least 2 additional toxins were detected in the fish sample by in vitro assay of liquid chromatography fractions. We cannot rule out the possibility that these toxins represent new ciguatoxinlike structures unique to the eastern Atlantic. Further studies are necessary to elucidate all toxins implicated in this outbreak.

Classic symptoms of ciguatera developed in our patients after eating a fish they captured in the Canary Islands, which are not in the ciguatera-endemic zone (Figure). The preliminary results of this outbreak investigation suggest the presence of ciguatoxins or ciguatoxinlike structures in fish from temperate waters of the eastern Atlantic. Ciguatera poisoning is a matter of public health concern and residents of coastal West Africa and the regional island archipelagos could be a new community at risk for this seafood intoxication syndrome. We emphasize that ciguatera poisoning is a debilitating disease, and therapeutic intervention strategies are very limited (*10*).
